# Facial Emphysema following Closure of Oroantral Fistulae

**DOI:** 10.1155/2021/5001266

**Published:** 2021-08-04

**Authors:** Muhammad Aiman Mohd Nizar, Syed Nabil

**Affiliations:** ^1^Department of Diagnostic and Bioscience Craniofacial, Faculty of Dentistry, Universiti Kebangsaan Malaysia, Malaysia; ^2^Department of Oral and Maxillofacial Surgery, Faculty of Dentistry, The National University of Malaysia, Malaysia

## Abstract

Subcutaneous emphysema (SE) is a swelling which develops due to air entrapped underneath the subcutaneous tissue and facial planes causing distention of the overlying skin. SE can develop due to trauma, surgery, or infection. The diagnosis of SE is mostly based on clinical findings of crepitation upon palpation of the swelling. Once diagnosed, SE is usually managed by close observation and in some cases may require surgical decompression and antibiotic prophylaxis. We report a rare case of SE of the left malar which developed following closure of oroantral communication using the buccal fat pad.

## 1. Introduction

Oroantral communication (OAC) is a condition where a connection exists between the maxillary sinus and the oral cavity, which, over time, will be lined by epithelium to form an oroantral fistula (OAF). The condition occurs commonly in upper posterior maxillary teeth extractions, especially ones with preexisting periapical abnormalities or when the roots are widely divergent [1]. Other reported causes of OAC include implant surgery, enucleation of cysts and tumors, maxillary orthognathic osteotomies, osteomyelitis, trauma, and various maxillary pathologic entities [1].

There are few treatment options for oroantral communication, including buccal advancement flap, plasma-rich fibrin and buccal fat pad (BFP), or a combination thereof. The BFP was mentioned for the first time by Heister in 1727 and was known as “glandula molaris” [2]. It was later described in detail by Marie-François-Xavier Bichat in 1802 [2]. It has been documented to provide a good clinical outcome when used for the closure of OAC [3]. Dolanmaz et al. reported that all of the 75 patients who underwent OAC closure with pedicled BFP in their series had successful outcomes [3].

Pedicled BFP is an established treatment option for OAF closure. Its richness of vascularity, low donor-site morbidity, simple surgical procedure, and proximity to the recipient site are the main advantages of this technique [4]. The familiar complications following this procedure include partial breakdown of the flap, especially in the closure of large maxillary defect, excessive scarring, and infection [5]. Emphysema, which develops as a result of air being pushed subcutaneously, is rarely anticipated with the use of BFP [6]. The authors report a rare case of subcutaneous emphysema at the left malar region that developed in the early postoperative period following closure of OAF using BFP.

## 2. Case Report

**Figure 1:**
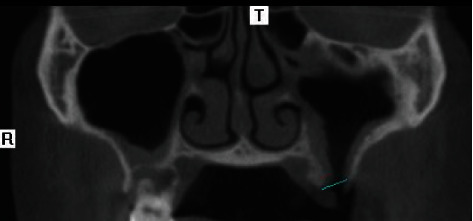
Cone beam computed tomography (CBTC) showing the oroantral communication in coronal view.

**Figure 2:**
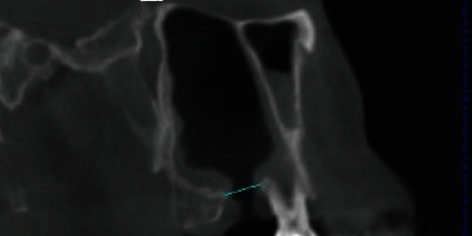
Cone beam computed tomography (CBTC) showing the oroantral communication in sagittal view.

A 45-year-old man visited our clinic with the complaint of a persistent hole in the 27-socket area. He explained that he had had tooth 27 extracted five years earlier and subsequently noted an opening at the socket which had never closed. He recalled that the extraction procedure itself had been uncomplicated. Subsequently, he had occasionally developed symptoms of chronic sinusitis such as nasal congestion and nasal discharge, but he had never sought treatment for it. There was also an occasional fluid discharge from the opening, but it never troubled him too much. Otherwise, he had no underlying medical illnesses. Upon examination, it was noted that there was a fistula of 1.5 cm × 1 cm in the area of the extracted 27. Tooth 16 was present, but 18 was missing. No fluid discharge was seen. Further investigation with cone beam CT confirmed an oroantral fistula measuring 9.4 mm × 7.7 mm (Figures 1 and 2). The sinus lining was noticeably thickened.

He underwent closure of the oroantral fistula under local anesthesia. A buccal mucoperiosteal flap was elevated around the fistula. Subsequently, the periosteum under the flap was incised to give access to the BFP. The BFP was mobilized and advanced to cover the fistula. Thereafter, the buccal mucoperiosteal flap was advanced over the BFP to achieve two-layered closure of the fistula. The advancing flaps were approximated with vertical mattress suturing. Bleeding was minimal throughout the procedure, and he was discharged with antibiotics, antihistamine, and nonsteroidal anti-inflammatory analgesia. He was advised on nasal precautions including instructions to sneeze with the mouth open and avoid blowing the nose, forceful spitting, and use of straws.

**Figure 3:**
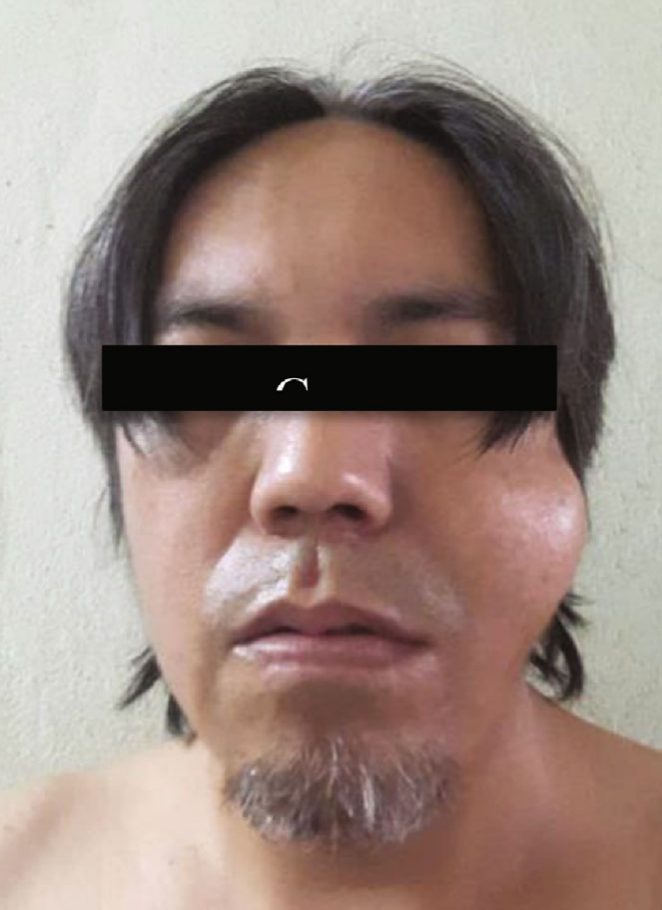
Immediately after swelling developed at the left malar region (around 2 hours postoperatively).

**Figure 4:**
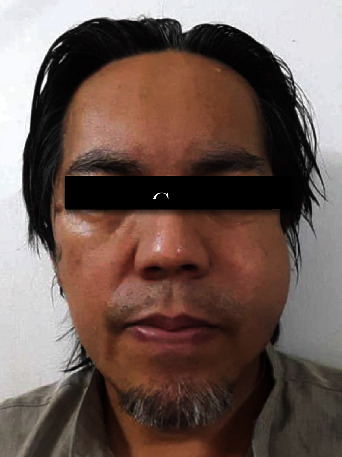
Postoperative 8 hours shows resolving of the left malar swelling.

Around 2 hours postoperative, he contacted the clinic to inform us that he had a sudden facial swelling that developed over a few seconds (Figure 3). The swelling occurred immediately after he tried to clear his throat. There was no associated pain or bleeding during the development of swelling. He was asked to come back to the clinic immediately. Upon examination of the swelling, crepitation was felt on palpation. Intraorally, the surgical wound was intact, and no indication of bleeding was noted. Diagnosis of emphysema was made on the basis of the sudden progression of swelling after a trigger and clinical findings. He was reassured, and reemphasis was placed on the sinus precaution. Besides the already prescribed medications, no additional intervention was made. The swelling was slightly reduced 8 hours after its initial appearance (Figure 4). Otherwise, he was well with minimal bleeding and pain.

**Figure 5:**
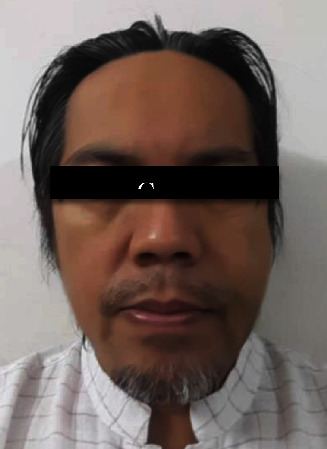
Postoperative day 3 shows that residual left malar swelling remains.

**Figure 6:**
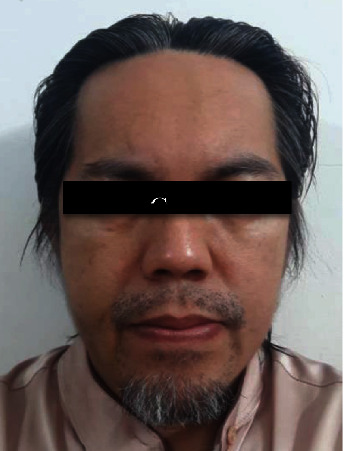
Postoperative day 7 shows that the swelling is completely resolved.

Upon review, 3 days later, the swelling was significantly reduced (Figure 5). At 7 days postoperative, the swelling had completely resolved (Figure 6). He was well throughout the postoperative period. Upon examination of the intraoral wound, no dehiscence was seen, and the oroantral fistula was completely closed. He was followed up again a few weeks later, and no reoccurrence of swelling or OAF was seen.

## 3. Discussion

Subcutaneous emphysema (SE) is a swelling that develops as a result of air being forced underneath the tissue and facial planes and is characterized by a crepitus sound upon palpation [7]. It could be of iatrogenic, incidental (patient factor), traumatic, or pathological cause [8]. It could be misinterpreted as a hypersensitivity reaction (angioedema), cellulitis, or necrotizing fasciitis [9]. In this case, the left malar SE developed following surgery using BFP for the closure of OAF when the patient tried to clear his throat. It is likely that the increase in intranasal pressure and subsequently in the maxillary sinus allowed air to escape through the sinus defect and travel to the subcutaneous tissue via the mucoperiosteal opening created to access the BFP. Roccia et al. explained that the increasing pressure inside the upper airways can lead the air to be forced into the surrounding tissues through mucoperiosteal tears resulting from the defects of the paranasal air sinuses and takes the path of least resistance between the loose connective tissues [10]. A study measuring the intranasal pressure in adults during nose blowing, sneezing, and coughing using fluid dynamic simulations concluded that the high intranasal pressures generated by nose blowing would propel viscous fluid into paranasal sinuses [11]. Therefore, it is possible that the act of clearing the throat with a closed mouth might also cause increased pressure in the paranasal sinuses.

SE usually is self-limiting and does not require treatment, but observation is crucial [8]. Occasionally, subcutaneous emphysema can cause infection [7, 12]. Antibiotics are therefore commonly prescribed in SE. The rationale for antibiotic prescription is that air introduced subcutaneously could be nonsterile [7]. A broad-spectrum-coverage antibiotic can be used to cover the more common head, neck, sinus, and skin microflora. Systemic steroids, meanwhile, can be used to reduce soft-tissue edema [13]. Rarely, an extensive SE may need multiple stab incisions to decompress the emphysema [8]. Subcutaneous emphysema could also be fatal in the case where the emphysema travels to the lateral pharyngeal space and may reach the mediastinum by dissecting the visceral space and causing pneumomediastinum, which is characterized by Hamman's sign—a friction rub sound upon cardiac auscultation [13]. In our reported case, the patient was prescribed antibiotics following surgery, and this was continued following the development of emphysema. However, no steroids were prescribed as the swelling was quite localized.

SE following a surgery of closure of the oroantral fistula using a pedicel BFP is very rare [14]. In the only previously reported case, swelling of the left cheek developed a few seconds after the patient sneezed two hours after the surgery. The swelling was associated with crepitus upon palpation. In that reported case, after a course of antibiotics, the emphysema completely resolved after 14 days. Similarly, in our case, two hours postoperative, a swelling developed at the left malar after the patient tried to clear his throat. Crepitation was felt on palpation of the swelling. We prescribed antibiotics, antihistamines, and nonsteroidal anti-inflammatory analgesia, and the swelling completely resolved after 10 days. Between these two cases, there are similarities in terms of onset, clinical presentation, management, and the final outcome. This suggests predictability in the causes of such occurrences, making prevention possible. More importantly, both had an uneventful recovery with conservative management; therefore, they should not discourage the clinician from utilizing BFP for similar OAF cases.

Here, we would like to emphasize that the postoperative instructions given to the patient should be very clear regarding the sinus precautions as it appears that in both cases, adherence to these instructions would have prevented the development of postoperative subcutaneous emphysema. These precautions include sneezing and coughing with the mouth open, avoiding blowing or whistling, and avoiding doing the Valsalva maneuver. Use of a straw and smoking are also prohibited. This will help to prevent an increase in nasal and intraoral pressure which may lead to subcutaneous emphysema. Strenuous physical activity, which will increase intrasinusoidal pressure, should also be avoided. Apart from that, there are options for medication that could reduce the risk of sneezing, such as antihistamines that will keep the patient's nasal dry and nasal decongestant.

In conclusion, subcutaneous emphysema is a possible complication following a closure of oroantral communication as reported here. This complication occurs due to air escaping the paranasal sinuses via an opening at the socket into the subcutaneous tissue of the malar area. The breached periosteum due to the harvesting of BFP is a crucial factor in allowing air to go subcutaneously. A crepitus sensation and sound upon palpation of the swelling is a pathognomonic feature of subcutaneous emphysema. Postoperative instructions should be explained very clearly to the patient to avoid this complication.

## Data Availability

Not applicable for this report.

## References

[1] Dym H., Wolf J. C. (2012). Oroantral communication. *Oral and maxillofacial surgery*.

[2] Marzano U. G. (2005). Lorenz Heister's ‘molar gland’. *Plastic and Reconstructive Surgery*.

[3] Dolanmaz D., Tuz H., Bayraktar S., Metin M., Erdem E., Baykul T. (2004). Use of pedicled buccal fat pad in the closure of oroantral communication: analysis of 75 cases. *Quintessence International (Berlin, Germany: 1985)*.

[4] Singh J., Prasad K., Lalitha R. M., Ranganath K. (2010). Buccal pad of fat and its applications in oral and maxillofacial surgery: a review of published literature (February) 2004 to (July) 2009. *Oral Surgery, Oral Medicine, Oral Pathology, Oral Radiology, and Endodontics*.

[5] Rapidis A. D., Alexandridis C. A., Eleftheriadis E., Angelopoulos A. P. (2000). The use of the buccal fat pad for reconstruction of oral defects: review of the literature and report of 15 cases. *Journal of Oral and Maxillofacial Surgery*.

[6] Hassani A., Shahmirzadi S., Saadat S. (2016). Applications of the buccal fat pad in oral and maxillofacial surgery. *A Textbook of Advanced Oral and Maxillofacial Surgery*.

[7] McKenzie W. S., Rosenberg M. (2009). Iatrogenic subcutaneous emphysema of dental and surgical origin: a literature review. *Journal of Oral and Maxillofacial Surgery*.

[8] Balaji S. M. (2015). Subcutaneous emphysema. *Journal of Maxillofacial and Oral Surgery*.

[9] Brasileiro B. F., Cortez A. L., Asprino L. (2005). Traumatic subcutaneous emphysema of the face associated with paranasal sinus fractures: a prospective study. *Journal of Oral and Maxillofacial Surgery: Official Journal of the American Association of Oral and Maxillofacial Surgeons*.

[10] Roccia F., Griffa A., Nasi A., Baragiotta N. (2003). Severe subcutaneous emphysema and pneumomediastinum associated with minor maxillofacial trauma. *The Journal of Craniofacial Surgery*.

[11] Gwaltney J. M., Hendley J. O., Phillips C. D., Bass C. R., Mygind N., Winther B. (2000). Nose blowing propels nasal fluid into the paranasal sinuses. *Clinical Infectious Diseases*.

[12] Shudo A. (2021). Buccal abscess derived from subcutaneous emphysema caused by the Valsalva maneuver after oral surgery with pedicled buccal fat pad grafting. *Oral Surgery*.

[13] Patel N., Lazow S. K., Berger J. (2010). Cervicofacial subcutaneous emphysema: case report and review of literature. *Journal of Oral and Maxillofacial Surgery*.

[14] Metin R., Tatli U. (2019). An unexpected complication after use of pedicled buccal fat pad for closure of oroantral fistulae: emphysema. *Journal of dentistry and oral sciences*.

